# Changes in Weight, Sedentary Behaviour and Physical Activity during the School Year and Summer Vacation

**DOI:** 10.3390/ijerph15050915

**Published:** 2018-05-04

**Authors:** Chiaki Tanaka, John J. Reilly, Maki Tanaka, Shigeho Tanaka

**Affiliations:** 1 Division of Integrated Sciences, J. F. Oberlin University, Tokyo 194-0294, Japan; 2 Physical Activity for Health Group, School of Psychological Sciences & Health, University of Strathclyde, Glasgow G1 1QE, UK; john.j.reilly@strath.ac.uk; 3 Department of Child Education, Kyoto Seibo College, Kyoto 612-0878, Japan; makit@jc.seibo.ac.jp; 4 Department of Nutrition and Metabolism, National Institute of Health and Nutrition, National Institutes of Biomedical Innovation, Health and Nutrition, Tokyo 162-8636, Japan; tanakas@nibiohn.go.jp

**Keywords:** adiposity, students, bidirectional

## Abstract

*Background*: To examine bidirectional associations between body weight and objectively assessed sedentary behaviour (SB) and physical activity (PA) during the school year and summer vacation. *Methods*: Participants were 209 Japanese boys and girls (9.0 ± 1.8 years at baseline). SB and PA were measured using triaxial accelerometry that discriminated between ambulatory and non-ambulatory PA, screen time measured by questionnaire during the school-term was evaluated in May and the summer vacation, and relative body weight measured in May and just after the end of summer vacation. *Results*: There were no significant relationships between changes in SB or PA and changes in body weight. However, higher relative body weight at baseline was associated with decreased non-ambulatory moderate PA (*p* = 0.049), but this association was slightly diminished after adjusting for change in SB (*p* = 0.056). Longer screen time at baseline was also associated with increased relative body weight (*p* = 0.033). *Conclusions*: The present study revealed that body weight might be particularly influential on non-ambulatory moderate PA while SB, PA or changes in these variables did not predict changes in body weight. Moreover, screen time during the school year is a predictor of change in relative body weight during the subsequent summer vacation.

## 1. Introduction

Prevalence of obesity has been decreasing in Japanese children recently according to a national survey [1], but remains much higher compared to 1980 or even 1990 [2]. A review found the most common seasonal pattern in six longitudinal descriptive studies in the USA and Japan was that children with obesity experienced accelerated gain in weight or body mass index (BMI) for age during the summer vacation, whereas healthy weight children gained less weight or maintained weight [3]. This review also suggested that physical activity (PA) may be the primary factor contributing to these seasonal (summer vacation) increases in weight or BMI, mainly because some studies indicated a decrease in PA in the summer vacation [3]. However, results on this issue have been inconsistent, and the cause-effect relationship has not been confirmed [3]. Thus, the impact of changes in PA, sedentary behaviour (SB), and weight status during the summer vacation is not fully understood. Moreover, the same review found that studies did not examine bidirectional associations between weight or weight status and daily SB and PA [3]. The benefits of regular moderate-to-vigorous intensity PA (MVPA) for obesity prevention are well acknowledged [4,5], but increased adiposity may also decrease PA [6]. Accelerometry as an objective measure of PA behaviour for children provides the full range of intensities of PA and SB; SB is distinct from PA [7,8,9], and it is possible for an individual to spend an excessive proportion of time in SB, even if they meet PA guidelines [10]. Some reviews [11,12,13] considered the association between objectively and subjectively measured SB and health outcomes in children and adolescents. Those reviews suggested that more evidence for cause-effect relationships between adiposity, SB, and PA is necessary.

Recently, Brazendale et al. reported that an unhealthy change in body composition during the summer could be explained by the “Structured Days Hypothesis” which posits that children engage in a greater number of unhealthy obesogenic behaviors (e.g., unfavorable activities/behaviors such as extended periods of sedentary/screen time and/or liberties to choose when, what, and how much to eat/drink) on unstructured days (e.g., summer vacation) when compared with structured days (school days) [14]. Weaver et al. also pointed out same hypothesis in their recent article [15].

In Japan, increased SB and decreased PA in summer vacation compared with those the school year in school children using accelerometry were reported [16]. However, it remains unclear as to whether measured habitual SB and PA changes are associated with weight gain, and whether any relationships are bidirectional in children across the school-term to summer vacation transition. Therefore, the reverse causation or ‘bidirectionality hypothesis’ needs to be tested. Thus, the main aim of the present study was to examine the longitudinal bidirectional associations between adiposity and daily SB and PA measured in Japanese school children, as a sub analysis of a previously published study [16]. Moreover, the determinants of change in body weight or measured changes in SB and PA or screen time were examined in the context of three potential determinants (eating habits, home environment and body image).

## 2. Materials and Methods

Our convenience sample included 209 Japanese primary children from four primary schools located in urban areas in Tokyo and Kyoto. Students and parents were invited to participate by leaflets at their school. Informed consent was obtained from all participants and their parents, and the Ethical Committee of J. F. Oberlin University approved the study protocol (No. 10007). Baseline data of anthropometric measurements, SB and PA were collected in May 2011 during the school year. The initial sample comprised *n* = 356 students. Due to missing data (no consent to take part/unable to trace for follow-up measures (*n* = 46), no accelerometer data at follow-up (*n* = 94), no height/weight data at follow-up (*n* = 7)), our longitudinal sample comprised data from 209 children. The follow-up data were collected in end of July or beginning of August during the summer vacation (mean interval, 64 SD 10 days). The anthropometric measurements were also measured just after the end of the summer vacation (mean interval, 114 SD 9 days).

### 2.1. Objective Measurement of Sedentary Behaviour and Physical Activity

Habitual SB, PA and step counts were measured with a triaxial accelerometer (Active style Pro HJA-350IT, Omron Healthcare, Kyoto, Japan), 74 × 46 × 34 mm and 60 g including batteries. Participants wore the accelerometer on the left side of the waist in May (school year) in the school year and July or August (summer vacation). In Japan, the “summer vacation” without classes in schools occurs for around 5 weeks from late July to late August. The details of the accelerometer are described elsewhere [17]. We calculated the synthetic acceleration of three axes using signals before and after high-pass filtering to remove the gravitational acceleration component from the signal using 10-s epoch. Then, the ratio of unfiltered to filtered acceleration was calculated and used to classify PA into ambulatory and non-ambulatory activities, in combination with synthetic acceleration itself. In our previous study, we reported the algorithm for the classification of daily life activities (Nintendo DS (Nintendo Inc., Kyoto, Japan), throwing a ball, household, etc.) and ambulatory activities by the unfiltered/filtered acceleration ratio, which resulted in 99.8% correct classification for eleven activities [17]. SB and PA were monitored continuously for 7 days or more. Participants were requested to wear these devices at all times, except under special circumstances, such as dressing and bathing. We accepted days in which more than 600 min (10 h) of wear time had accrued, not counting time allowed for the above-mentioned unavoidable reasons. In addition, periods with over 60 min of consecutive ‘‘non-wear time’’ were considered to be non-wear time. The criteria used in the present study were similar to those in other papers [18]. The 2 weekdays plus 1 weekend day represented a minimum required for inclusion. In practice, the amount of accelerometry obtained was much greater than this. Previous studies suggested at least 3 days were required for reliable PA monitoring in young children [19,20]. Participants with data from at least 2 weekdays and at least 1 weekend day in the school years and at least 3 days in the summer vacation were included in the analysis, because they went to their school on neither weekdays nor weekend days in the summer vacation and there was no large difference between weekdays and weekend days. The inclusion/exclusion criteria used in the present study were similar to those in other papers [16,18]. PA consists of ambulatory activity such as walking and running and non-ambulatory activity such as performing playing with blocks, tossing a ball, playing games, aerobic dance and household activities (e.g., typing, vacuuming, dishwashing, and fidgeting). Crouter et al. also proposed an algorithm to classify daily living activities into continuous walking/running and intermittent lifestyle activities in children [21]. In fact, our previous study showed that non-ambulatory time as measured by triaxial accelerometry was much longer than ambulatory time during medium-intensity PA in free-living Japanese preschool children [22]. Furthermore, step counts were also measured by the accelerometer.

### 2.2. Anthropometric Measurements

Body height and weight were measured to the nearest 0.1 cm and 0.1 kg, respectively. Height and body weight was measured without shoes, but with clothing. Net body weight was calculated as the weight of clothing subtracted from the measured body weight. Clothing for each participant included prescript shorts and t-shirt, corresponding to a 0.5 kg deduction. BMI was calculated as weight in kilograms divided by height in meters squared. Weight status was classified as normal weight, overweight/obesity, or thin based on Japanese cut-offs for weight status that were established based on national reference data for Japanese children [23]. Relative body weight was used as an index of weight status [23], calculated as follows:

Relative body weight = [measured body weight (kg) − standard weight for gender, age, and height(kg)]/standard weight (kg) × 100 (%)

※ standard weight (kg) = a × measured body height (cm) − b

a and b are gender- and age-specific.

The cut-offs of weight states are as follows: Overweight/Obesity combined: ≥+20%, Normal weight: −20% to + 20% and Thinness: ≤−20% [23].

### 2.3. Self-Reported Measures and Interview

Since participants were young, questionnaire data were collected from participants answering with their parents following the methods of the Japanese national health and nutrition examination survey. The type of sedentary behaviour was assessed by two questions; “How many hours do you/your child usually watch of television (TV) or video movies per day on weekday and weekend, respectively?, and “How many hours do you/your child usually play games (including games on TV, personal computer (PC), handheld game console etc.) or use the PC per day on weekday and weekend, respectively? A systematic review [24] found a dearth of high-quality evidence on the determinants of measured SB and PA in children and adolescents. Therefore, the present study included four of the categories of determinant derived from a socio-ecological model as recommended, except for a social-cultural environmental domain [24,25,26]. The variables studied for each domain were:

(a) Demographic and biological domain: gender; age.
(b) A psychological domain: body image.
(c) A behavioural domain: eating habits.
(d) A physical environmental domain: television set in children’s bedroom; availability of air conditioner during summer vacation.

Participants and their parents were asked about eating habits; habitual frequency of having a snack at daytime and night, having a snack at night and during daytime with television viewing at baseline and follow-up and having a breakfast and dinner with television viewing at baseline, the volume of having juice including sports drinks, vegetables and fruits juice at follow-up. Participants and their parents were also asked about bedroom television ownership. Body image perception was asked for each participant by an interview.

### 2.4. Statistical Analysis

The average time at SB and each PA intensity per day was calculated by METs recorded every 10 sec as follows: average number of weekday and weekend minutes spent in SB (METs ≤ 1.5), LPA (1.6 ≤ METs < 3.0), MVPA (METs ≥ 3.0), moderate PA (MPA) (3.0 ≤ METs < 6.0) and vigorous PA (VPA) (6.0 ≤ METs) were calculated for each individual, and then average weekly values were calculated. For the data during the school year, average values were calculated by weighting for 5 weekdays and 2 weekend days (Weighted data = ((average for weekdays × 5) + (average for weekend days × 2))/7). PA assessed by the accelerometer is presented as: (1) PA states for ambulatory activity or non-ambulatory activity in intensity-specific categories (LPA, MVPA, MPA and VPA); and (2) number of steps registered per day.

Values of SB and PA were adjusted for wear time. Change variables were calculated as follow-up values minus baseline values. There were no significant interactions between gender and seasonal variation of each variable. A paired t-test was used to compare baseline and follow-up measurements. Partial correlations were analyzed between SB and each PA at baseline adjusted for gender, school, age and body height at baseline. Partial correlations were analyzed also between change in SB and each PA adjusted for gender, school, follow-up period, age and body height at baseline.

The associations between change in body weight or relative body weight and SB, PA or screen time at baseline variables were analyzed by analysis of covariance (ANCOVA) adjusted for gender, school, follow-up period, age, body weight, body height, and SB or PA at baseline. The associations between change of SB or PA and relative body weight at baseline variables were analyzed by ANCOVA adjusted for gender, school, follow-up period, age, relative body weight and SB or PA at baseline. Moreover, when the result of each first analysis was significant, in the final stage of the analysis, other SB or PA variables were also adjusted in the same model to evaluate an independent effect of SB or PA at baseline. The reverse (bidirectional) associations were analyzed by the same modelling procedure. For analysis of questionnaire data, total screen time included TV viewing, PC use, and video game play. When the number of answers for behavioural variables was below 10, the category was added to another category. The associations between change in body weight, relative body weight, and accelerometer data were analyzed by ANCOVA. The covariates were the same as the analyses described above. The associations between weight status at baseline (overweight/obese children versus normal weight children) and change of body weight, SB or PA or relative body weight were analyzed by ANCOVA adjusted for gender, school and age at baseline, follow-up period, and change of body height, SB or PA at baseline. Statistical analyses were performed with IBM SPSS statistics 20.0 for Windows (IBM Co., Tokyo, Japan). All statistical tests were regarded as significant when *p*-values were less than 0.05.

## 3. Results

### 3.1. Characteristics of Study Participants

The participants’ characteristics are shown in Table 1. Age, body height and body weight significantly increased from baseline to follow-up. The numbers of overweight/obesity, normal-weight and thin participants were 12 (5.7%), 194 (92.8%) and 3 (1.4%) at baseline and 16 (7.7%), 189 (90.4%) and 4 (1.9%) at just after summer vacation, respectively. The duration of accelerometry was much greater than the minimum criteria specified (at least 3 days and 10 h), with an average of 7.1 days and 13.4 h per day at baseline and 8.0 days and 12.8 h per day at follow-up, respectively. Ambulatory and total time in LPA, MVPA, MPA and VPA, non-ambulatory time in VPA and step counts significantly decreased from baseline to follow-up. Measured SB and screen time and non-ambulatory time in MPA significantly increased. The partial correlations at baseline or the change between SB and LPA were strong (r = −0.953 and r = −0.958, *p* < 0.001). The partial correlations between SB and MVPA (r = −0.626 and r = −0.665, *p* < 0.001) or MPA (r = −0.664 and r = −0.691, *p* < 0.001) were moderate. The partial correlations between SB and VPA (r = −0.273, *p* < 0.001 and r = −0.258, *p* = 0.001) were significant but weak. There were no correlations between SB and each screen time.

### 3.2. Baseline Body Weight as a Predictor of Change in Sedentary Behaviour, Physical Activity, and Vice Versa

Relative body weight at baseline was significantly associated with the change in non-ambulatory MPA (Table 2). This association was slightly diminished after adjusting for change in measured SB. Longer screen time at baseline was associated with increase in relative body weight. Changes in body weight or relative body weight were not associated with change in evaluated various intensity PAs or SB or screen time (Table 3).

### 3.3. Influence of Baseline Overweight and Obesity on Changes in Sedentary Behaviour and Physical Activity

Weight status (overweight/obesity vs normal weight children) and change in body weight or change in SB or PA were not related significantly. The changes of relative body weight from baseline were −3.3% in the children with overweight/obesity children and −1.3% in the normal weight children, respectively. The relative body weight decreased significantly more in children with overweight/obesity than in normal weight children (*p* = 0.035). In addition, weight status of most participants did not change, while 4 normal-weight participants became overweight, 2 normal-weight participants became thin, and a thin participant became normal-weight.

### 3.4. Determinants of Change of Body Weight, Relative Body Weight, Sedentary Behaviour or Physical Activity

Results of the analyses are shown in Table 4 and Table 5. Results of eating measures and frequency of air conditioner use are shown in Supplementary Table S1. The body image by self-interview was significantly associated with increased change of relative body weight adjusted for relative body weight at baseline. Bedroom television ownership was significantly associated with increased change of SB or decreased change of PA adjusted for SB or PA at baseline. Other variables by the questionnaire or interview were not associated with body weight, relative body weight, SB or PA variables.

## 4. Discussion

In the present study, the relative body weight at baseline was significantly associated with the change in non-ambulatory MPA (β = −0.060, *p* = 0.049), but not vice versa. For primary school children, non-ambulatory activities include playing games, radio gymnastics, tossing a ball, cleaning and clearing away, bedmaking, dressing and undressing, etc. [17,27]. Moreover, the screen time at baseline was associated with change in relative body weight (β = −0.009, *p* = 0.033), but not vice versa. On the other hand, there were no association between the relative body weight at baseline and change in LPA or VPA. It is not clear why no association was observed for LPA such as normal walking [17,27]. Time in VPA such as jogging might be too short to have significant associations [17,27].

There are only a few studies which have comparatively evaluated PA between summer vacation and the school year, using accelerometry or the doubly labelled water method [28,29]. However, the results of these studies were not consistent. Moreover, these previous studies did not examine bidirectional associations between weight and SB or PA. As mentioned above, a previous review suggested that PA is the primary factor contributing to seasonal (summer vacation) increases in weight or weight status but there is no evidence to what extent weight or weight status at school term to affect PA or SB in summer vacation [3]. The present study suggested that body weight might be particularly influential on non-ambulatory MPA (*p* = 0.049), but this association was slightly diminished after adjusting the change in measured SB (*p* = 0.056) while SB, PAs or changes in these variables did not predict changes in adiposity. Screen time during the school year may also be a predictor of change in relative body weight during the subsequent summer vacation. We identified only four prospective studies that examined bidirectional associations between adiposity and measured by accelerometer SB and/or PA in children and adolescents [30,31,32,33], although they examined bidirectional associations over longer periods (one or two year). The present study findings are consistent with the previous studies in primary school children [32,33] indicating that low PA may be a result rather than a cause of adiposity. The differences in age between the present study and two others previous studies for younger children and adolescents may also account, in part, for differences in the findings between the present study and some previous studies [32,33].

Changes in factors other than PA and SB may be important influences on subsequent adiposity. Longer screen time at baseline was associated with increase in relative body weight in the present study. A review reported that higher durations/frequencies of screen time and TV viewing were associated with unfavourable body composition [34]. In the present study, the association between longer TV viewing at baseline and an increase in relative body weight was almost significant while that of game using time at baseline was not. In other countries, sedentary behavior guidelines are devised separately from physical activity guidelines [35,36,37]. These guidelines state that school-age children and adolescents should spend no more than 2 h per day in recreational screen time. Mean screen time at baseline and follow-up were over 2 h per day in the present study. Setting a limit to the time spent watching TV might be supported by the present study, but it is likely that screen-based games do not represent such a high risk compared to watching TV. Another review reported that the potential mediators of the effect of higher TV viewing on higher BMI include less time for PA, increased energy intake (from more eating while watching TV and a greater exposure to marketing of energy dense foods) in children and adolescents [34]. The Report of National Survey on Physical Fitness, Athletic Performance and Exercise Habits of the Japan Sports Agency reported that 73% of grade 5 Japanese primary school children spent more than 1 h per day of watching TV, videos or DVDs viewing (Not playing video games) [38]. Thus, a setting a limit to the time spent watching TV will be an important possibility in preventing an increase in relative body weight for Japanese children. Moreover, bedroom television at baseline was associated with increasing in SB or decreasing in PA. Saelens et al. [39] reported that having a TV set in the bedroom were longitudinally associated with TV viewing time at ages 6 and 12 years, as well as with increases in TV viewing at these ages. Previous studies reported that overweight Japanese children experienced greater weight gain during the summer than during school months [40,41]. The trend of accelerated summer weight gain in overweight children has been also reported by several researchers from other countries, and PA is suggested as the primary factor contributing to these seasonal (summer vacation) increases in body weight [3]. In the present study, only relative body weight at baseline was significantly associated with the change in non-ambulatory MPA. However, except for that, weight status, SB or PA at baseline changes were not correlated. One of the possible reasons for lack of associations between some variables in the present study was that the participants of the present study showed summer weight gains which were too small (Table 1, body weight: +1.1 kg) to detect such associations. Moreover, relative body weight decreased significantly more in overweight/obese children than normal weight children, which is different from results of some of previous reports [3].

Previous studies proposed the “Structured Days Hypothesis” which posits that children engage in a greater number of unhealthy obesogenic behaviors (e.g., unfavorable activities/behaviors such as extended periods of sedentary/screen time and/or liberties to choose when, what, and how much to eat/drink) on unstructured days (e.g., summer vacation) when compared with structured days (in school year). In the present study, eating habits were not associated with change in relative weight. One of the reasons might be limited questions about eating habits when compared with previous studies [14,15].

There were several limitations in the current study. The convenience sample may be not representative of the population. Moreover, only a small percentage of the sample was overweight/obese (5.7% at baseline) in the present study. However, the School Health Survey data in 2011 by the Ministry of Education, Culture, Sports, Science and Technology reported that 3.84% to 8.81% of Japanese primary school children were overweight/obese [42]. According to the School Health Survey data, mean values of height and body weight were 130.8 cm and 29.0 kg, respectively. Children in the present study were 131.8 ± 11.7 cm and 29.3 ± 8.1 kg at baseline. Thus, body size of the study sample was comparable with Japanese population. As a consequence, it may not be appropriate to extend our results to obese populations. The sample size may have limited our ability to examine the impact of weight status on subsequent behaviour in the school holiday. These were growing children and fluctuations in body weight were normal (e.g., growth spurts). Actually, the length of time between baseline and follow-up was relatively short, because an average period of summer vacation in primary school is about 35 days in Japan, which are decided by local government and because the present study focused on the weight gain during the summer vacation. The accelerometer used in the present study is widely used to measure PA as it has an excellent ability to measure various types of PA, but may not accurately assess all types of PA, such as swimming and cycling. We made no direct measures of body composition in the present study. Questionnaire data were collected from participants answering with their parents following the methods of the Japanese National Health and Nutrition Examination Survey except for body image perception. The eating habits and body image perception were qualitatively and poorly assessed, since validated tools in children were not used, such as those to assess dietary habits [43] and body image [44]. Another limitation is that there were almost 1.5 months difference in timing of the measurements for the summer vacation between PAs and SB or questionnaire measurements and the anthropometric measurements. Body weight and height were measured just after the summer vacation. However, SB and PAs may have changed in some participants, and as a result, the periods from the baseline to follow-up for weight and SB and PAs are different. Nonetheless, to our knowledge, this study is the first study to explicitly examine the reverse causation hypothesis using the summer vacation period. However, future studies should prospectively examine the bidirectional association between adiposity and patterns of SB in more participants with larger variability to clarify the cause-effect relationship, considering the above limitations, particularly important as populations continue to increase in adiposity.

## 5. Conclusions

In conclusion, the present study suggested that the children with low body weight or low relative body weight at baseline showed smaller increases in SB and smaller decreases in PA during the summer vacation than those with higher body weight or relative body weight at baseline. The present study also suggests that higher body weight or relative body weight might be particularly influential on decreasing non-ambulatory MPA. Moreover, the longer screen time and distorted body image perception in the school year is a predictor of change in increasing relative body weight during the subsequent summer vacation. Bedroom television ownership may be a predictor of change in increasing SB or decreasing PA during the subsequent summer vacation. More evidence on the relationship between weight status and life habits during summer vacation is needed, and guideline development for summer vacation for children might be necessary. A school education programme prior to summer holidays about screen time and an environment at home, and student’s body image also might benefit children’s health.

## Figures and Tables

**Table 1:** Physical characteristics, habitual sedentary behaviour and physical activity, screen time, bedroom television ownership and body image for participants.

	Baseline			Follow-Up			*p* Value
*n* = 209	Mean	±	SD	Mean	±	SD	
Age (years) *	9.0	±	1.8	9.3	±	1.8	<0.001
Height (cm) *	131.8	±	11.7	134.2	±	12.0	<0.001
Body weight (kg) *	29.3	±	8.1	30.5	±	8.5	<0.001
BMI (kg/m^2^) *	16.6	±	2.3	16.6	±	2.3	0.388
Relative weight (%) *	−0.9	±	12.3	−1.9	±	12.5	<0.001
Weight status (Overweight and obesity: %) *	5.7	±		7.7	±		
Sedentary behaviour (min/day)	346.2	±	64.4	365.8	±	72.1	<0.001
LPA (min/day)							
Ambulatory	102.6	±	18.4	93.0	±	24.7	<0.001
Non-ambulatory	258.1	±	45.4	254.7	±	46.3	0.176
Total time	360.7	±	52.0	347.7	±	56.8	<0.001
MVPA (min/day)							
Ambulatory	38.3	±	14.2	31.1	±	16.9	<0.001
Non-ambulatory	27.6	±	8.5	28.4	±	9.2	0.059
Total time	65.8	±	19.9	59.4	±	22.8	<0.001
MPA (min/day)							
Ambulatory	33.8	±	12.2	28.3	±	14.9	<0.001
Non-ambulatory	26.0	±	8.0	27.2	±	8.8	0.004
Total time	59.7	±	17.3	55.5	±	20.1	<0.001
VPA (min/day)							
Ambulatory	4.5	±	2.6	2.7	±	2.5	<0.001
Non-ambulatory	1.6	±	1.3	1.2	±	1.4	<0.001
Total time	6.0	±	3.6	3.9	±	3.7	<0.001
Step count (steps/day)	10931	±	2450	9462	±	3287	<0.001
TV time (min/day) (*n* = 175)	109.9	±	64.4	119.9	±	76.8	0.014
Game time (min/day) (*n* = 175)	26.2	±	31.1	33.1	±	41.5	0.002
Screen time (min/day) (*n* = 175)	136.1	±	80.7	153.0	±	97.0	0.001
	*n*	%					
Bedroom television ownership	205	98					
1．yes	46	22					
2．no	159	76					
Body image perception of participants	209						
1．considerably obese	3	1					
2．slightly obese	23	11					
3．maintain the present body	155	74					
4．slightly thin	22	11					
5．considerably thin	6	3					

BMI: body mass index, LPA: Light physical activity, MVPA: moderate-to-vigorous physical activity, MPA: moderate physical activity, VPA: vigorous physical activity, *: follow-up was just after summer vacation.

**Table:** (**a**)

	Outcome: ΔBody Weight (kg)					Exposure: Body Weight at Baseline (kg)			
	β-Coefficiet	95% CI		*p*-Value		β-Coefficient	95% CI		*p*-Value
Sedentary behaviour at baseline (min/day)	0.001	−0.002	0.004	0.667	ΔSedentary behaviour (min/day)	1.004	−0.741	2.748	0.258
LPA at baseline (min/day)					ΔLPA (min/day)				
Ambulatory	−0.004	−0.013	0.006	0.444	Ambulatory	−0.512	−1.193	0.170	0.141
Non−ambulatory	0.000	−0.004	0.004	0.942	Non-ambulatory	−0.550	−1.725	0.624	0.357
Total time	−0.001	−0.004	0.003	0.714	Total time	−1.199	−2.635	0.236	0.101
MVPA at baseline (min/day)					ΔMVPA (min/day)				
Ambulatory	−0.006	−0.020	0.008	0.415	Ambulatory	0.092	−0.339	0.522	0.675
Non-ambulatory	0.011	−0.011	0.032	0.323	Non-ambulatory	−0.119	−0.315	0.077	0.233
Total time	−0.001	−0.011	0.009	0.873	Total time	0.014	−0.500	0.527	0.958
MPA at baseline (min/day)					ΔMPA (min/day)				
Ambulatory	−0.008	−0.024	0.008	0.349	Ambulatory	0.123	−0.259	0.505	0.527
Non-ambulatory	0.010	−0.013	0.032	0.400	Non-ambulatory	−0.144	−0.333	0.044	0.133
Total time	−0.001	−0.013	0.010	0.815	Total time	−0.022	−0.477	0.434	0.926
VPA at baseline (min/day)					ΔVPA (min/day)				
Ambulatory	−0.012	−0.082	0.057	0.728	Ambulatory	0.025	−0.016	0.066	0.224
Non-ambulatory	0.087	−0.047	0.222	0.203	Non-ambulatory	0.024	−0.013	0.061	0.204
Total time	0.005	−0.044	0.055	0.828	Total time	0.033	−0.062	0.128	0.496
Step count at baseline (steps/day)	0.000	0.000	0.000	0.145	ΔStep count (steps/day)	−7.278	−97.320	82.765	0.874
TV time at baseline (min/day)	0.002	−0.001	0.005	0.180	ΔTV time (min/day)	0.457	−1.664	2.577	0.671
Game time at baseline (min/day)	0.005	−0.001	0.011	0.083	ΔGame time (min/day)	−0.916	−1.981	0.149	0.091
Screen time at baseline (min/day)	0.002	0.000	0.004	0.076	ΔScreen time (min/day)	−0.463	−2.922	1.996	0.711

LPA: light physical activity, MVPA: moderate-to-vigorous physical activity, MPA: moderate physical activity, VPA: vigorous physical activity, Δ: change, Δvariables were calculated as follow-up values minus baseline values, adjusted for gender, school, age and body height at baseline, follow-up period, sedentary behaviour or physical activity and body weight at baseline.

**Table:** (**b**)

	Outcome: ΔRelative Body Weight (%)					Exposure: Relative Body Weight at Baseline (%)			
	β-Coefficient	95% CI		*p*-Value		β-Coefficient	95% CI		*p*-Value
Sedentary behaviour at baseline (min/day)	0.006	−0.005	0.016	0.311	ΔSedentary behaviour (min/day)	0.367	−0.190	0.924	0.195
LPA at baseline (min/day)					ΔLPA (min/day)				
Ambulatory	0.001	−0.032	0.034	0.939	Ambulatory	−0.125	−0.340	0.091	0.257
Non-ambulatory	−0.010	−0.025	0.005	0.171	Non-ambulatory	−0.240	−0.612	0.132	0.205
Total time	−0.008	−0.021	0.005	0.241	Total time	−0.428	−0.886	0.029	0.066
MVPA at baseline (min/day)					ΔMVPA (min/day)				
Ambulatory	−0.010	−0.059	0.040	0.702	Ambulatory	0.022	−0.114	0.159	0.749
Non-ambulatory	0.028	−0.046	0.103	0.454	Non-ambulatory	−0.056	−0.118	0.007	0.080
Total time	0.001	−0.034	0.036	0.965	Total time	−0.025	−0.188	0.139	0.766
MPA at baseline (min/day)					ΔMPA (min/day)				
Ambulatory	−0.010	−0.067	0.048	0.743	Ambulatory	0.030	−0.092	0.151	0.630
Non-ambulatory	0.028	−0.052	0.108	0.498	Non-ambulatory	−0.060	−0.120	0.000	0.049
					*Non-ambulatory	−0.058	−0.118	0.002	0.056
Total time	0.002	−0.038	0.043	0.911	Total time	−0.044	−0.106	0.017	0.158
VPA at baseline (min/day)					ΔVPA (min/day)				
Ambulatory	−0.094	−0.341	0.152	0.451	Ambulatory	0.000	−0.021	0.022	0.967
Non-ambulatory	0.177	−0.300	0.654	0.466	Non-ambulatory	0.004	−0.007	0.016	0.461
Total time	−0.024	−0.200	0.152	0.788	Total time	0.005	−0.025	0.035	0.738
Step count at baseline (steps/day)	0.000	0.000	0.000	0.492	ΔStep count (steps/day)	−5.766	−34.025	22.493	0.688
TV time at baseline (min/day)	0.010	0.000	0.020	0.060	ΔTV time (min/day)	0.095	−0.602	0.792	0.788
Game time at baseline (min/day)	0.016	−0.005	0.037	0.128	ΔGame time (min/day)	−0.133	−0.483	0.217	0.455
Screen time at baseline (min/day)	0.009	0.001	0.017	0.033	ΔScreen time (min/day)	−0.035	−0.844	0.773	0.932

LPA: light physical activity, MVPA: moderate-to-vigorous physical activity, MPA: moderate physical activity, VPA: vigorous physical activity, Δ: change, Δvariables were calculated as follow-up values minus baseline values, adjusted for gender, school, age at baseline, follow-up period, sedentary behaviour or physical activity and relative body weight at baseline, *: adjusted for Δsedentary behaviour.

**Table 3:** Associations between change on body weight or relative weight and change on sedentary behaviour or physical activity.

	Exposure: ΔBody Weight (kg)				Exposure: ΔRelative Body Weight (%)			
	β-Coefficient	95% CI		*p*-Value	β-Coefficient	95% CI		*p*-Value
ΔSedentary behaviour (min/day)	0.001	−0.002	0.004	0.471	0.004	−0.007	0.015	0.516
ΔLPA (min/day)								
Ambulatory	−0.003	−0.011	0.005	0.492	−0.013	−0.041	0.016	0.382
Non-ambulatory	−0.001	−0.006	0.003	0.571	−0.001	−0.018	0.015	0.872
Total time	−0.001	0.587	−0.005	−0.544	−0.002	−0.015	0.011	0.771
ΔMVPA (min/day)								
Ambulatory	−0.001	−0.014	0.012	0.834	−0.012	−0.058	0.034	0.597
Non-ambulatory	−0.004	−0.031	0.022	0.743	−0.035	−0.130	0.060	0.473
Total time	−0.001	−0.012	0.009	0.814	−0.013	−0.051	0.024	0.485
ΔMPA (min/day)								
Ambulatory	−0.001	−0.016	0.014	0.894	−0.018	−0.070	0.034	0.490
Non-ambulatory	−0.002	−0.030	0.026	0.875	−0.033	−0.132	0.066	0.514
Total time	−0.001	−0.013	0.011	0.859	−0.018	−0.061	0.024	0.397
ΔVPA (min/day)								
Ambulatory	−0.002	−0.073	0.069	0.964	0.078	−0.175	0.332	0.542
Non-ambulatory	−0.062	−0.199	0.074	0.368	−0.119	−0.607	0.370	0.632
Total time	−0.011	−0.064	0.043	0.699	0.027	−0.165	0.218	0.784
ΔStep count (steps/day)	0.000	0.000	0.000	0.502	0.000	0.000	0.000	0.427
ΔTV time at baseline (min/day)	0.001	−0.002	0.004	0.584	−0.004	−0.015	0.008	0.512
ΔGame time at baseline (min/day)	−0.004	−0.010	0.003	0.262	−0.012	−0.035	0.011	0.307
ΔScreen time at baseline (min/day)	0.000	−0.003	0.003	0.981	−0.005	−0.015	0.005	0.313

LPA: light physical activity, MVPA: moderate-to-vigorous physical activity, MPA: moderate physical activity, VPA: vigorous physical activity, Δ: change, Δvariables were calculated as follow-up values minus baseline values, adjusted for gender, school, age at baseline, body height (just analysis of Δbody weight) at baseline, follow-up period, Δsedentary behaviour, Δphysical activity, Δbody weight or Δrelative body weight.

**Table 4:** Associations between change of relative weight and body image at baseline.

Adjusted Variables	Body Image Perception of Participants	Dependent Variable: Change of Relative Body Weight (1: *n* = 26, 2: *n* = 155, 3: *n* = 28)				Dependent Variables	Adjusted body Weight at Baseline (1: *n* = 26, 2: *n* = 155, 3: *n* = 28)				Adjusted Relative Body Weight at Baseline (1: *n* = 26, 2: *n* = 155, 3: *n* = 28)			
		Estimated	SE	B	*p*-Value		Estimated	SE	B	*p*-Value	Estimated	SE	B	*p*-Value
		Mean					Mean				Mean			
Sedentary behaviour at baseline (min/day)	1. considerably obesity/slightly obesity	1.3	1.0	3.3	0.024 (1 VS 2)	ΔSedentary behaviour (min/day)	19.7	12.5	7.1		25.1	12.3	11.9	
	2. maintain the present body	−1.5	0.4	0.6			13.4	4.4	0.8		13.2	4.5	−0.1	
	3. slightly thin/considerably thin	−2.0	0.8	0.0	0.045 (1 VS 3)		12.6	10.1	0.0		13.2	10.3	0.0	
* Sedentary behaviour at baseline (min/day)	1. considerably obesity/slightly obesity	1.4	1.0	3.5	0.016 (1 VS 2)									
	2. maintain the present body	−1.5	0.4	0.6										
	3. slightly thin/considerably thin	−2.1	0.8	0.0	0.032 (1 VS 3)									
LPA at baseline (min/day)						ΔLPA (min/day)								
Ambulatory	1. considerably obesity/slightly obesity	1.3	1.0	3.4	0.018 (1 VS 2)	Ambulatory	−4.2	4.8	5.3		−7.3	4.7	1.8	
	2. maintain the present body	−1.5	0.4	0.5			−7.8	1.7	1.8		−7.7	1.8	1.4	
	3. slightly thin/considerably thin	−2.0	0.8	0.0	0.039 (1 VS 3)		−9.5	4.0	0.0		−9.1	4.0	0.0	
* Ambulatory	1. considerably obesity/slightly obesity	1.2	1.0	3.2	0.028 (1 VS 2)									
	2. maintain the present body	−1.5	0.4	0.5										
	3. slightly thin/considerably thin	−2.0	0.8	0.0										
Non-ambulatory	1. considerably obesity/slightly obesity	1.2	1.0	3.3	0.024 (1 VS 3)	Non-ambulatory	−13.0	8.3	−15.0		−13.8	8.1	−15.0	
	2. maintain the present body	−1.5	0.4	0.6			−1.9	3.0	−3.9		−1.8	3.0	−3.0	
	3. slightly thin/considerably thin	−2.0	0.8	0.0	0.046 (1 VS 3)		2.0	6.8	0.0		1.2	6.8	0.0	
* Non-ambulatory	1. considerably obesity/slightly obesity	1.2	1.0	3.3	0.022 (1 VS 2)									
	2. maintain the present body	−1.5	0.4	0.5										
	3. slightly thin/considerably thin	−2.1	0.8	0.0	0.044 (1 VS 3)									
Total	1. considerably obesity/slightly obesity	1.3	1.0	3.3	0.023 (1 VS 2)	Total	−18.5	10.2	−10.9		−21.6	10.0	−13.4	
	2. maintain the present body	−1.5	0.4	0.6			−9.2	3.6	−1.6		−9.0	3.7	−0.8	
	3. slightly thin/considerably thin	−2.0	0.8	0.0	0.043 (1 VS 3)		−7.6	8.3	0.0		−8.2	8.4	0.0	
* Total	1. considerably obesity/slightly obesity	1.3	1.0	3.4	0.018 (1 VS 2)									
	2. maintain the present body	−1.5	0.4	0.6										
	3. slightly thin/considerably thin	−2.1	0.8	0.0	0.035 (1 VS 3)									
MVPA at baseline (min/day)						ΔMVPA (min/day)								
Ambulatory	1. considerably obesity/slightly obesity	1.3	1.0	3.4	0.019 (1 VS 2)	Ambulatory	−1.8	3.0	5.7		−3.4	3.0	4.1	
	2. maintain the present body	−1.5	0.4	0.6			−5.1	1.1	2.4		−5.0	1.1	2.5	
	3. slightly thin/considerably thin	−2.0	0.8	0.0	0.040 (1 VS 3)		−7.5	2.5	0.0		−7.5	2.5	0.0	
* Ambulatory	1. considerably obesity/slightly obesity	1.3	1.0	3.4	0.022 (1 VS 2)									
	2. maintain the present body	−1.5	0.4	0.6										
	3. slightly thin/considerably thin	−2.1	0.8	0.0	0.041 (1 VS 2)									
Non-ambulatory	1. considerably obesity/slightly obesity	1.4	1.0	3.5	0.013 (1 VS 2)	Non-ambulatory	−1.3	1.4	−4.2		−1.4	1.4	−4.1	
	2. maintain the present body	−1.5	0.4	0.5			1.6	0.5	−1.2		1.7	0.5	−1.0	
	3. slightly thin/considerably thin	−2.1	0.8	0.0	0.031 (1 VS 2)		2.9	1.1	0.0		2.6	1.1	0.0	
* Non-ambulatory	1. considerably obesity/slightly obesity	1.3	1.0	3.4	0.017 (1 VS 2)									
	2. maintain the present body	−1.5	0.4	0.5										
	3. slightly thin/considerably thin	−2.1	0.8	0.0	0.036 (1 VS 2)									
Total	1. considerably obesity/slightly obesity	1.4	1.0	3.5	0.015 (1 VS 2)	Total	−3.1	3.7	1.5		−4.6	3.6	0.3	
	2. maintain the present body	−1.5	0.4	0.5			−3.5	1.3	1.1		−3.4	1.3	1.5	
	3. slightly thin/considerably thin	−2.1	0.8	0.0	0.034 (1 VS 3)		−4.6	3.0	0.0		−4.9	3.0	0.0	
* Total	1. considerably obesity/slightly obesity	1.4	1.0	3.5	0.016 (1 VS 2)									
	2. maintain the present body	-1.5	0.4	0.6										
	3. slightly thin/considerably thin	−2.1	0.8	0.0	0.032 (1 VS 3)									
MPA at baseline (min/day)						ΔMPA (min/day)								
Ambulatory	1. considerably obesity/slightly obesity	1.3	1.0	3.4	0.019 (1 VS 2)	Ambulatory	−1.0	2.7	4.6		−2.4	2.6	3.3	
	2. maintain the present body	−1.5	0.4	0.6			−3.8	1.0	1.8		−3.7	1.0	2.0	
	3. slightly thin/considerably thin	−2.0	0.8	0.0	0.040 (1 VS 3)		−5.6	2.2	0.0		−5.7	2.2	0.0	
* Ambulatory	1. considerably obesity/slightly obesity	1.3	1.0	3.4	0.022 (1 VS 2)									
	2. maintain the present body	−1.5	0.4	0.6										
	3. slightly thin/considerably thin	−2.1	0.8	0.0	0.041 (1 VS 3)									
Non-ambulatory	1. considerably obesity/slightly obesity	1.4	1.0	3.4	0.014 (1 VS 2)	Non-ambulatory	−1.1	1.3	−4.3		−1.3	1.3	−4.2	
	2. maintain the present body	−1.5	0.4	0.5			1.9	0.5	−1.2		2.0	0.5	−1.0	
	3. slightly thin/considerably thin	−2.1	0.8	0.0	0.033 (1 VS 3)		3.1	1.1	0.0		2.9	1.1	0.0	
* Non-ambulatory	1. considerably obesity/slightly obesity	1.3	1.0	3.4	0.020 (1 VS 2)									
	2. maintain the present body	−1.5	0.4	0.6										
	3. slightly thin/considerably thin	−2.1	0.8	0.0	0.038 (1 VS 3)									
Total	1. considerably obesity/slightly obesity	1.4	1.0	3.5	0.015 (1 VS 2)	Total	−2.1	3.2	0.4		−3.7	3.2	−0.9	
	2. maintain the present body	−1.5	0.4	0.6			−1.9	1.2	0.6		−1.8	1.2	1.0	
	3. slightly thin/considerably thin	−2.1	0.8	0.0	0.033 (1 VS 3)		−2.5	2.6	0.0		−2.8	2.7	0.0	
* Total	1. considerably obesity/slightly obesity	1.3	1.0	3.5	0.016 (1 VS 2)									
	2. maintain the present body	−1.5	0.4	0.6										
	3. slightly thin/considerably thin	−2.1	0.8	0.0	0.031 (1 VS 3)									
VPA at baseline (min/day)						ΔVPA (min/day)								
Ambulatory	1. considerably obesity/slightly obesity	1.3	1.0	3.3	0.024 (1 VS 2)	Ambulatory	−1.1	0.5	0.7		−1.2	0.5	0.6	
	2. maintain the present body	−1.5	0.4	0.6			−1.2	0.2	0.6		−1.2	0.2	0.6	
	3. slightly thin/considerably thin	−2.0	0.8	0.0	0.044 (1 VS 3)		−1.8	0.4	0.0		−1.8	0.4	0.0	
* Ambulatory	1. considerably obesity/slightly obesity	1.2	1.0	3.3	0.029 (1 VS 2)									
	2. maintain the present body	−1.4	0.4	0.6										
	3. slightly thin/considerably thin	−2.0	0.8	0.0	0.049 (1 VS 3)									
Non-ambulatory	1. considerably obesity/slightly obesity	1.4	1.0	3.4	0.010 (1 VS 2)	Non-ambulatory	−0.2	0.3	0.1		−0.2	0.3	0.1	
	2. maintain the present body	−1.6	0.4	0.4			−0.2	0.1	0.0		−0.2	0.1	0.1	
	3. slightly thin/considerably thin	−2.0	0.8	0.0	0.032 (1 VS 3)		−0.3	0.2	0.0		−0.3	0.2	0.0	
* Non-ambulatory	1. considerably obesity/slightly obesity	1.4	1.0	3.4	0.014 (1 VS 2)									
	2. maintain the present body	−1.6	0.4	0.4										
	3. slightly thin/considerably thin	−2.0	0.8	0.0	0.039 (1 VS 3)									
Total	1. considerably obesity/slightly obesity	1.4	1.0	3.4	0.019 (1 VS 2)	Total	−1.2	0.7	0.9		−1.2	0.7	0.9	
	2. maintain the present body	−1.5	0.4	0.5			−1.5	0.2	0.6		−1.5	0.2	0.6	
	3. slightly thin/considerably thin	−2.0	0.8	0.0	0.038 (1 VS 3)		−2.1	0.6	0.0		−2.1	0.6	0.0	
* Total	1. considerably obesity/slightly obesity	1.3	1.0	3.3	0.023 (1 VS 2)									
	2. maintain the present body	−1.5	0.4	0.5										
	3. slightly thin/considerably thin	−2.0	0.8	0.0	0.043 (1 VS 3)									
Step counts at baseline (steps/day)	1. considerably obesity/slightly obesity	1.3	1.0	3.3	0.021 (1 VS 2)	ΔStep counts (steps/day)	−851.3	633.3	555.6		−983.1	620.1	466.2	
	2. maintain the present body	−1.4	0.4	0.6			−1076.4	231.6	330.5		−1072.8	232.2	376.5	
	3. slightly thin/considerably thin	−2.0	0.8	0.0	0.040 (1 VS 3)		−1406.9	520.2	0.0		−1449.4	521.8	0.0	
TV time at baseline (min/day)	1. considerably obesity/slightly obesity	2.1	1.0	4.0	0.004 (1 VS 2)	ΔTV time (min/day)	−31.5	13.7	−43.7	0.010 (1 VS 2)	−33.6	13.4	−45.4	0.005 (1 VS 2)
	2. maintain the present body	−1.4	0.4	0.5			11.6	4.9	−0.6		11.9	5.0	0.1	0.047 (2 VS 3)
	3. slightly thin/considerably thin	−1.9	0.9	0.0	0.017 (1 VS 3)		12.2	11.5	0.0		11.8	11.6	0.0	
Game time at baseline (min/day)	1. considerably obesity/slightly obesity	2.0	1.0	4.0	0.005 (1 VS 2)	ΔGame time (min/day)	12.9	7.2	6.5		7.2	7.0	−0.9	
	2. maintain the present body	−1.5	0.4	0.5			9.6	2.6	3.2		9.7	2.6	1.6	
	3. slightly thin/considerably thin	−1.9	0.9	0.0	0.019 (1 VS 3)		6.4	6.0	0.0		8.1	6.0	0.0	
Screen time at baseline (min/day)	1. considerably obesity/slightly obesity	2.0	1.0	3.9	0.006 (1 VS 2)	ΔScreen time (min/day)	−19.9	16.2	−38.8		−27.7	15.7	−47.9	0.011 (1 VS 2)
	2. maintain the present body	−1.4	0.4	0.4			21.0	5.8	2.1		21.5	5.8	1.2	
	3. slightly thin/considerably thin	−1.9	0.9	0.0	0.023 (1 VS 3)		18.9	13.5	0.0		20.3	13.6	0.0	

LPA: Light physical activity, MVPA: moderate-to-vigorous physical activity, MPA: moderate physical activity, VPA: vigorous physical activity, Δ: change, Δvariables were calculated as follow-up values minus baseline values, adjusted for gender, school, follow-up periods, age, relative body weight and sedentary behavior, physical activity or TV time, Game time or screen time at baseline, *: adjusted by sedentary behaviour or physical activity at baseline.

**Table 5:** Associations between change of sedentary behaviour or physical activity and bedroom television ownership.

Dependent Variables	Ownership of Television at the Participant‘s Bedroom	Adjusted Body Weight at Baseline				Adjusted Body Relative Body Weight at Baseline			
		Estimated Mean	SE	B	*p*-Value	Estimated Mean	SE	B	*p*-Value
ΔSedentary behaviour (min/day)	Yes	27.4	7.2	17.7	0.022	28.6	7.2	18.9	0.015
	No	9.6	4.1	0.0		9.7	4.1	0.0	
ΔLPA (min/day)									
Ambulatory	Yes	−10.4	3.0	−3.3	0.298	−10.8	3.0	−3.7	0.244
	No	−7.1	1.7	0.0		−7.1	1.7	0.0	
Non-ambulatory	Yes	−9.9	5.0	−10.1	0.060	−10.4	5.0	−10.6	0.049
	No	0.2	2.9	0.0		0.2	2.9	0.0	
Total	Yes	−19.7	6.1	−13.3	0.041	−20.6	6.1	−14.2	0.030
	No	−6.4	3.5	0.0		−6.4	3.5	0.0	
ΔMVPA (min/day)									
Ambulatory	Yes	−7.8	1.8	−3.3	0.090	−8.0	1.8	−3.5	0.074
	No	−4.6	1.0	0.0		−4.5	1.0	0.0	
Non-ambulatory	Yes	0.6	0.8	−1.2	0.172	0.5	0.8	−1.4	0.134
	No	1.8	0.5	0.0		1.8	0.5	0.0	
Total	Yes	−7.3	2.2	−4.5	0.055	−7.6	2.2	−4.8	0.042
	No	−2.8	1.3	0.0		−2.8	1.3	0.0	
ΔMPA (min/day)									
Ambulatory	Yes	−6.4	1.6	−3.2	0.062	−6.6	1.6	−3.4	0.050
	No	−3.2	0.9	0.0		−3.2	0.9	0.0	
Non-ambulatory	Yes	0.9	0.8	−1.2	0.181	0.8	0.8	−1.3	0.140
	No	2.1	0.5	0.0		2.1	0.5	0.0	
Total	Yes	−5.5	1.9	−4.3	0.036	−5.8	1.9	−4.6	0.026
	No	−1.2	1.1	0.0		−1.2	1.1	0.0	
ΔVPA (min/day)									
Ambulatory	Yes	−1.4	0.3	−0.2	0.580	−1.4	0.3	−0.2	0.552
	No	−1.3	0.2	0.0		−1.3	0.2	0.0	
Non−ambulatory	Yes	−0.4	0.2	−0.1	0.544	−0.4	0.2	−0.1	0.533
	No	−0.2	0.1	0.0		−0.2	0.1	0.0	
Total	Yes	−1.8	0.4	−0.7	0.053	−1.8	0.4	−0.3	0.555
	No	−1.5	0.2	0.0		−1.5	0.2	0.0	
ΔStep count (steps/day)	Yes	−1469.9	388.4	−441.1	0.290	−1496.8	386.6	−467.4	0.261
	No	−1028.7	225.0	0.0		−1029.5	225.0	0.0	

LPA: Light physical activity, MVPA: moderate-to-vigorous physical activity, MPA: moderate physical activity, VPA: vigorous physical activity, Δ: change, Δvariables were calculated as follow-up values minus baseline values, adjusted for gender, school, follow-up periods, age, body weight or relative body weight and sedentary behaviour or physical activity at baseline.

## Supplementary Materials

The following are available online at http://www.mdpi.com/1660-4601/15/5/915/s1, Table S1: Eating habits and frequency of air conditioner use for participants.
