# Supplementary material for: Changes in Weight, Sedentary Behaviour and Physical Activity during the School Year and Summer Vacation
Source: Int J Environ Res Public Health. 2018 May 4;15(5):915. doi: 10.3390/ijerph15050915 (PMC5981954; doi:10.3390/ijerph15050915)
Supplement: Supplementary file 1 [file ijerph-15-00915-s001.pdf]

Table S1 Eating habits and frequency of air conditioner use for participants

	Baseline		Follow-up	
	n	%	n	%
Having a snack during daytime	205	98		
1. almost every day	116	56		
2. 4-5 days/week	40	19		
3. 2-3 days/week	35	17		
4. eating seldom	14	7		
Having a snack at night	205	98		
1. almost every day	14	7		
2. 4-5 days/week	11	5		
3. 2-3 days/week	23	11		
4. eating seldom	157	75		
Having a snack at daytime during television viewin	206	99		
1. normally	48	23		
2. sometimes	44	21		
3. rarely	60	29		
4. never	54	26		
Having a breakfast during television viewing	206	99		
1. normally	96	46		
2. sometimes	22	11		
3. rarely	30	14		
4. never	58	28		
Having a dinner during television viewing	206	99		
1. normally	85	41		
2. sometimes	36	17		
3. rarely	34	16		
4. never	51	24		
Volume of having juice			204	98
1. more than 4 cups of juice/day			4	2
2. 2-3 cups of juice/day			49	23
3. a cup of juice/day			61	29
4. less than a cup of juice/day			90	43
Frequency of air conditioner use			205	98
1. normally			15	7
2. sometimes			82	39
3. rarely			64	31
4. never			44	21
